# Mr. Hornbrook, on Ol. Tereb. in Burns

**Published:** 1805-07-01

**Authors:** John Hornbrook

**Affiliations:** Tavistock


### *To the Editors of the Medical and Physical Journal.*

GENTLEMEN,

IF the annexed case should be deemed worthy of being inserted in your useful Journal, it is hoped it will be a means of proving the superior efficacy of Mr. Kentish's practice in burns. Your's respectfully,

*Tavistock, June* 5, 1805. JOHN HORNBROOK.

---

SAMUEL RUNDLE, aged forty-two, labourer at a brewery in Tavistock, passing over the mash tub on a plank, slipt his foot, and fell into the boiling mash up to his knees. When

When I saw him, his situation was truly lamentable; in taking off his stockings, the cuticle, which had been totally detached by the boiling water, was lacerated and torn in ribands; he was held by two of his fellow labourers, in order to prevent his dancing about the room, in consequence of the excruciating pain; the whole of his legs were immediately washed with ol. tereb. and afterwards dressed with a liniment made of ung. basil. flav. and ol. tereb.; a glass of strong brandy and water with opii pur. gr. ij. were immediately given, and a mixture with tinct. opii camph. to be taken every two hours, with orders that he might take any thing comfortable either to eat or drink.

Second day, The pain gradually abated after he was dressed, for about three hours, when it remained stationary and returned in paroxysms, which became less painful and of shorter duration the one than the other. The parts were washed with ol. tereb. and the former dressings applied; the mixture continued and the pil ex opio at night; the generous diet to be persevered in as at first ordered.

Third day, Has passed a tolerable easy night; pulse 100; some appearance of secretion where the vesications were. Cease to apply the ol. tereb. and liniment to these parts, and use the cerat. é. lapid. calaminar. The diet was gradually diminished in respect to its stimulant qualities; the proportion of the fermented liquors less; calomel, gr. vj. opii, gr. j. cons. ros. q. s. f. bol. h. s. s. as he is rather costive.

Fourth, fifth, and sixth days, he continued to do well; pus secreted where there were vesications, and the process of skinning is going on rapidly on both legs. Continue the cerate, and the diet to be now moderate.

Eighth day, He walked to the brewery and back again without my knowledge or consent.

From the eighth to the seventeenth day, nothing particular took place to retard the progress of the cure, which, in respect to the scald, might be said to be compleat, as the legs were now covered with new skin; to guard which from cracking, I ordered the liniment with ol. lini. and aq. calcis to be applied with a feather.

The liniment with ol. lini. and aq. calcis answer very well in this stage of the complaint, as an application to harden the new skin and to prevent its cracking, as new cicatrices are liable to.

Nineteenth day and regularly since, he has followed his usual employ in the brewery.

 A similar

A similar accident happened to one of the partners in the same brewery, about three years since; the treatment having been totally different, he was three months before he left his room. I am induced, in justice to Mr. Kentish's Essay on Burns, to publish this case, to prove the advantages derived from his treatment as being far superior to any I ever knew employed.

